# Targeting lymphatic dysfunction in atherosclerosis: a state-of-the-art review on potential therapies and future directions

**DOI:** 10.3389/fcvm.2026.1774468

**Published:** 2026-03-18

**Authors:** Andrea Varias-Menor, Annalise Michlin, Brandon Stere, Sharif Afifi, Vasiliki Tasouli-Drakou, John Varras

**Affiliations:** Department of Internal Medicine, Kirk Kerkorian School of Medicine at University of Nevada Las Vegas, Las Vegas, NV, United States

**Keywords:** apolipoprotein A-I therapy, atherosclerosis, inflammation and immune regulation, lymphagiogenesis, lymphatic dysfunction, lymphatic-targeted therapeutics, nanoparticle-based gene delivery, plaque stability

## Abstract

Atherosclerosis remains a leading cause of cardiovascular morbidity and mortality worldwide. While traditionally attributed to lipid accumulation, endothelial dysfunction, and inflammation, growing evidence implicates the lymphatic system as a key regulator of vascular homeostasis and plaque stability. Recent experimental data suggest that restoring lymphatic function may represent a novel therapeutic avenue in atherosclerosis. Recombinant VEGF-C variants and nanoparticle-based gene delivery systems selectively have been shown to induce lymphangiogenesis and improve lipid clearance without triggering abnormal angiogenesis. Similarly, Apolipoprotein A-I infusions have been demonstrated to strengthen lymphatic endothelial junctions, enhance vessel contractility, and facilitate the removal of cholesterol and inflammatory cells from atherosclerotic lesions. This comprehensive review aims to present recent findings from preclinical and clinical trials and studies on investigational pharmacological therapies, explore the interrelationship between atherosclerosis and the lymphatic system, and highlight potential avenues for future research.

## Highlights

1. Restoring lymphatic function represents a novel therapeutic paradigm for plaque and inflammation resolution in atherosclerosis.
2. Future therapies targeting VEGF-C/VEGFR3 signaling may improve lymphatic drainage and promote reverse cholesterol transport.

## 1 Introduction

Atherosclerosis is a chronic arterial disease characterized by the accumulation of atheromatous plaques within the arterial walls. This condition accounts for a significant proportion of mortality in Western societies, contributing to a substantial number of deaths attributed to myocardial infarction, stroke, and peripheral arterial disease (1). For decades, atherosclerosis research has focused on endothelial injury, foam cell formation, and plaque development as central pathological events (2). However, growing evidence suggests that the lymphatic system may play a crucial role in the progression and resolution of atherosclerosis.

Recent studies suggest that perivascular lymphatic vessels within the arterial wall play a key role in removing lipids, inflammatory substances, and immune cells from atherosclerotic lesions (3, 4). Therefore, when lymphatic drainage function is compromised, it has been linked to accelerated lesion development and increased inflammation (5, 6). Recently, emerging therapies for atherosclerosis have been focusing on enhancing lymphatic function to combat vascular disease. These include therapies that target the Vascular Endothelial Growth Factor-C (VEGF-C) and the Vascular Endothelial Growth Factor Receptor 3 (VEGFR3) to promote lymphangiogenesis in cardiac dysfunction to therapeutic modalities that involve administering Apolipoprotein A-I (ApoA-I) to reverse cholesterol transport and improve lymphatic flow (7, 8). More specifically, VEGF-C plays a crucial role in reverse cholesterol transport and immunomodulation, helping reduce plaque size and macrophage expression in atherosclerosis.

**Figure 1:**
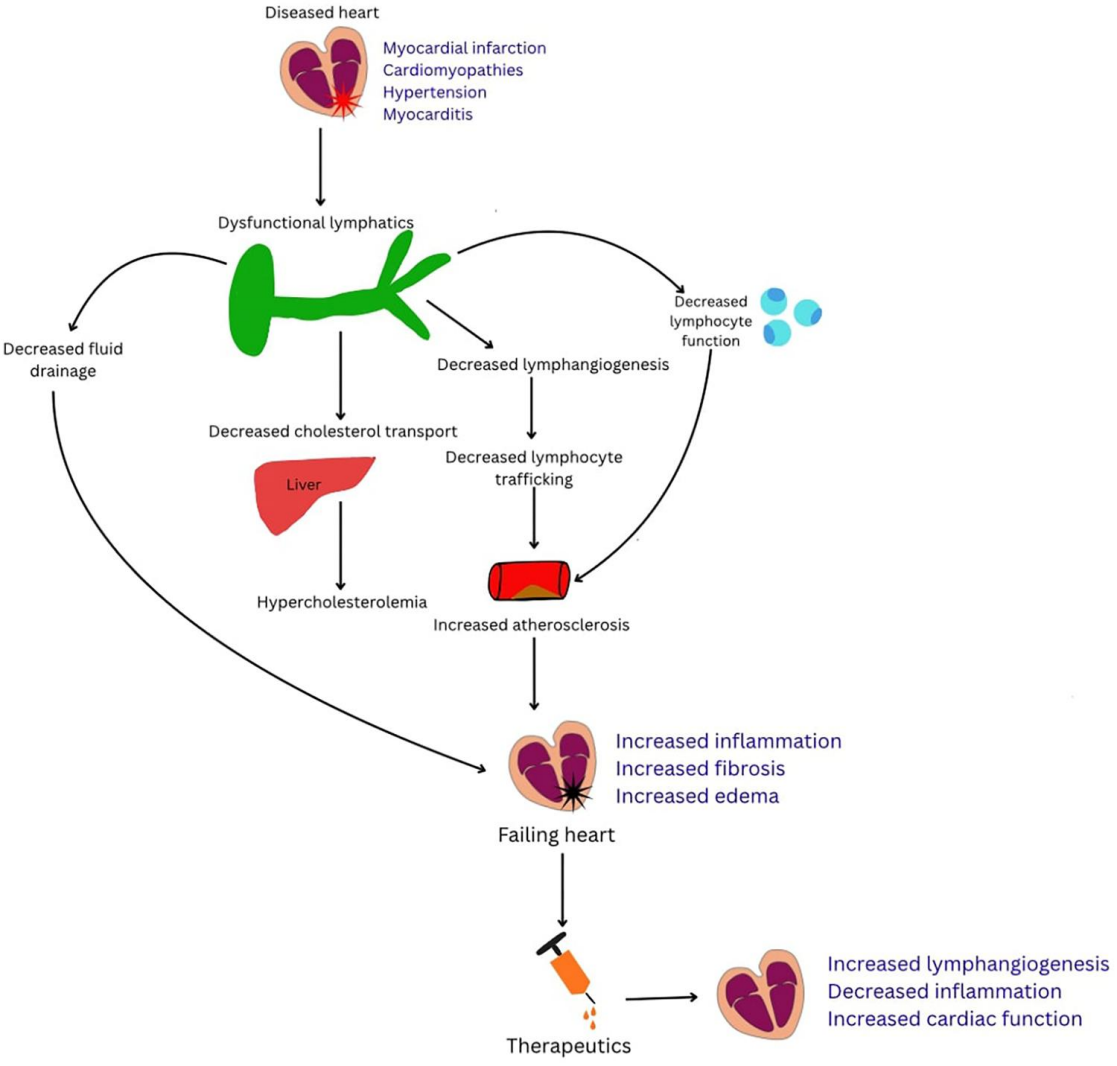
Figure summarizing how cardiac injury drives lymphatic dysfunction and contributes to atherosclerosis progression. Diseased cardiac states—including myocardial infarction, cardiomyopathies, hypertension, and myocarditis—lead to dysfunctional lymphatics with impaired fluid drainage, decreased lymphangiogenesis, reduced cholesterol transport, and diminished lymphocyte trafficking. These abnormalities promote hypercholesterolemia, persistent inflammation, and accelerated atherosclerosis, ultimately worsening cardiac failure. Lymphangiogenic therapeutics aim to restore lymphatic function, reduce inflammation, and improve cardiac performance.

Nonetheless, despite advances over recent years, critical gaps remain in our understanding of how lymphatic dysfunction contributes to atherosclerotic plaque progression and instability. Likewise, the therapeutic potential of enhancing lymphatic function to reduce plaque burden in humans remains largely unexplored. This review article seeks to summarize the pathophysiology of and interrelationship between atherosclerosis and the lymphatic system, explore investigational pharmacological therapies, and highlight potential avenues for future research (Figure 1).

## 2 Materials and methods

A literature search was conducted across multiple bibliographic databases, including PubMed, MEDLINE, and Google Scholar. Medical Subject Headings (MeSH) terms used in the search included “lymphatic system”, “atherosclerosis”, and “inflammation”. Systematic reviews, meta-analyses, preclinical and clinical studies published in the English language from January 1, 2000 to December 1, 2025 that provided insight into the relationship between lymphatic dysfunction, atherosclerosis and inflammation (two of them or all three) were selected. Additionally, clinical trials assessing pharmacological interventions aimed at enhancing lymphatic function and their effects on atherosclerosis were reviewed. No case studies were evaluated to produce this comprehensive review. All the sources were evaluated for relevance, credibility, and peer-review status. Gaps in the current literature were identified to highlight areas for future research.

## 3 Pathophysiology

The lymphatic system is a component of both the immune and circulatory systems, functioning as a network composed of lymph nodes, lymphatic vessels, lymphatic cells, and multiple organs including the spleen and thymus. Its primary responsibility most known is to preserve fluid homeostasis throughout the vessels within the body and the interstitial tissues around them. This is done through the lymphatic vessels normally seen running alongside major blood vessels and within a majority of organs (9). The smallest vessels within the lymphatic system are capillaries with thin walls less than 100 micrometers in diameter, made up of a single layer of endothelial cells (10). The layers possess junctions along their basement membranes and anchoring filaments to maintain an opening within the lumen (11). These junctions and filaments keep the lymphatic capillaries highly permeable, which in turn allow them to collect fluid and solutes from within the interstitial tissues. The lymphatic capillaries are unidirectional and take the fluid absorbed from within the tissues to larger collecting lymphatic vessels. The large collecting vessels are composed of an endothelial layer composed of many tight junctions that form a characteristic “zipper-like” pattern (12).

Lymphatic vessels are classified as afferent when they deliver fluid from the interstitial tissues to local lymph nodes, and efferent when they deliver the fluid from lymph nodes to subsequent lymph nodes, and ultimately the venous system (13). The network of larger vessels the capillaries deliver fluid to ultimately converges to form two major lymphatic channels, the right lymphatic duct and the thoracic duct. The right lymphatic duct is responsible for draining interstitial fluid located in the right upper quadrant of the chest, the right upper extremity, and the head (9). The remaining lymphatic channels in the body lead to the thoracic duct, which is generally the largest lymphatic vessel.

### 3.1 Atherosclerosis & the role of inflammation in atherosclerosis

A function of the lymphatic system that has become an increasingly interesting area of research is its interaction with cardiovascular health, specifically in regards to regulating the development of atherosclerotic plaques. Though traditionally associated with comorbidities like hyperlipidemia, hypertension, smoking, and so forth, there is now data suggesting that a dysregulation of the lymphatic system can enhance and even facilitate the formation of atherosclerotic plaque (2). Before discussing the link between the lymphatic system and atherosclerotic plaques, the pathophysiology of plaque formation, and the role the lymphatic system plays in regulating inflammation must first be determined. Atherosclerosis is generally understood to be an arterial disease, characterized by the deposition of plaques onto their inner walls. These plaques are comprised of fatty acids, cholesterol, calcium, and a fibrous cap (14). Over time, the buildup of the plaque causes the arterial walls to become more rigid, allowing subsequent plaques to grow and occlude the lumen of the arteries (15). This in turn increases the risk of plaque rupture and ischemic events in which oxygen-saturated blood cannot be effectively delivered to dependent tissue.

Endothelial injury is proposed to be an early pathophysiologic event in the formation of atherosclerotic plaques (16). When it presents within regions of arterial vasculature, it is the earliest detectable change in the timeline of the development of an atherosclerotic lesion. Triggers predisposing to endothelial injury include known cardiovascular risk factors like hypertension, hypercholesterolemia, diabetes, and smoking (17). These factors can damage the endothelium through a variety of mechanisms including damaging the endothelial cells directly, increasing the levels of low-density lipoprotein (LDL), and generating an imbalance between free radicals and antioxidants. The infiltration of LDLs into the tunica intima of endothelial cells, a process that is mediated by scavenger receptor B1 (SR-B1) and activin A receptor-like type 1 (ALK1) receptor, triggers the recruitment of monocytes and their transformation into M1 macrophages in the subendothelial space (14). These macrophages internalize the trapped lipoproteins to become foam cells (18).

**Figure 2:**
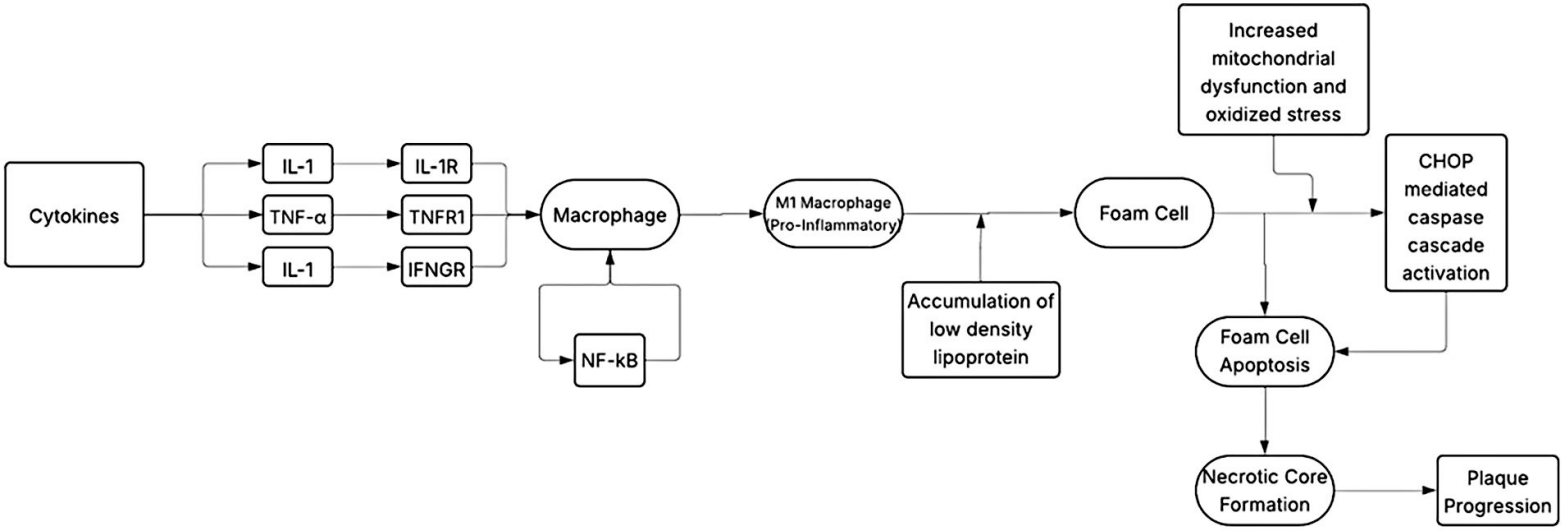
A schematic diagram on the genesis of atherosclerotic plaque development through the activation of pro-inflammatory cytokines.

**Figure 3:**
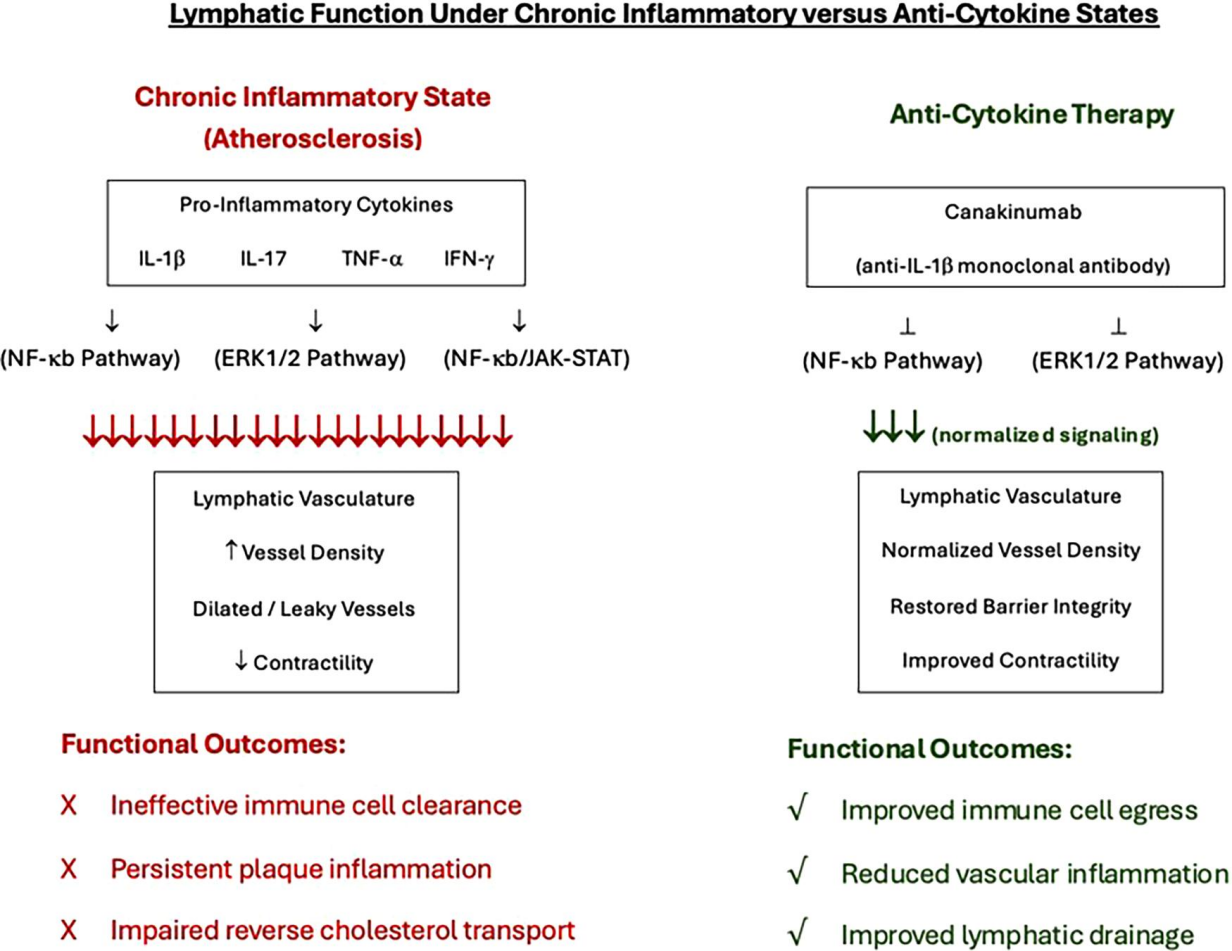
A schematic on the balance between inflammatory cytokine-driven lymphangiogenesis and anti-cytokine therapy in atherosclerosis.

The loss in functionality of the endothelium reduces nitric oxide (NO) levels and subsequently is associated with an increased expression of proinflammatory adhesion molecules, cytokines, and chemotactic factors (18). Low concentrations of NO lead to a loss in the inhibition of cytokine and chemokine synthesis, leukocyte adhesion, and transmigration (19). Chemokines Monocyte Chemoattractant Protein-1 (MCP-1), C-X3-C motif ligand (CX3CL) 1 and C-C motif ligand (CCL) 5 are associated with monocyte recruitment, while adhesion molecules vascular cellular adhesion molecule (VCAM)-1 and intercellular adhesion molecule (ICAM)-1 allow the monocytes to migrate into the tunica intima by binding to their specific receptors on monocytes (20). Multiple pro-inflammatory cytokines are expressed during the development and progression of atherosclerosis. Cytokine interferon gamma (IFN-γ) induces the expression of M1 macrophages through the JAK-STAT pathway and contributes to the growing atherosclerotic plaque size by inducing foam cell apoptosis and the release of lipid protein into core of the plaque. Other cytokines include the tissue necrosis factor alpha (TNF-α) which upregulates the expression of MCP-1, ICAM-1 and VCAM-1, Interleukin-6 (IL-6) which promotes fatty streaks and induces the release of TNF-α, IL-18 which upregulates IFN-γ production, and IL-1α and IL-1β which regulate the activation of macrophages (21). Th17 lymphocytes further produce IL-21 and IL-22, which play an important role in the accumulation of macrophages. The expression of these cytokines is controlled by the activation of nuclear factor-kappa B (NF-κB) whose expression is upregulated in atherosclerosis (Figure 2). Through a positive feedback loop, the expression of one pro-inflammatory cytokine induces the activation and production of another. Figure 3 provides a schematic on how anti-cytokine therapy attenuates excessive, dysfunctional lymphangiogenesis while restoring net lymphatic function in chronic vascular inflammation.

### 3.2 The connection between the lymphatic system and inflammation

The lymphatic system plays an important role in mediating inflammatory responses. This is accomplished through its responsibility in draining extravasated fluid, leukocytes, and inflammatory mediators (22, 23). One key mediator of inflammation is VEGF-C, a protein crucial for lymphangiogenesis through its binding to receptor VEGFR3 and loosely to receptor VEGFR2 following its cleavage by enzyme ADAMTS3 (24). Through tyrosine autophosphorylation of the cytoplasmic tail of its receptors, VEGF-C induces the formation of VEGFR2-VEGFR3 heterodimers or VEGFR3 homodimers (25). VEGFR-3 homodimers are implicated in the organization of endothelial cells and lumen formation, while VEGFR2-VEGFR3 heterodimers contribute to angiogenic sprouting.

VEGF-C expression may show distinct functional and perhaps opposing effects when its expression is analyzed in an acute setting against a chronic counterpart. In an acute phase, inflamed tissues show an increase in the release of VEGF-C by immune cells including dendritic cells, neutrophils, and macrophages, with its expression primarily upregulated in response to proinflammatory cytokines (26). For example, cytokine IL-17 has been found to upregulate VEGF-C expression through the activation of the ERK 1/2 pathway (25). Cytokines IFN-γ and TNF-α have also been shown to upregulate VEGF-C expression, especially in studies exploring wound healing using mesenchymal cells (27). Likewise, cytokine IL-1β has been proven to upregulate VEGF-C expression through the NF-κB pathway. Of note, IL-1β and TNF-α have been shown specifically to increase VEGF-C production through increasing its mRNA transcription rather than through stabilizing existing strands, demonstrated in a study utilizing a nuclear run-on method (28). While not directly linked to VEGF-C, cytokines IL-6 and IL-8, have also been linked to increased VEGF expression (29).

On the other hand, VEGF-C expression may also be increased in more chronic pro-inflammatory states. For example, its increased expression is observed in a variety of cancers, such as lung, breast, ovarian and bladder cancers, in the latter of which the carcinoembryonic antigen-related cell adhesion molecule 1 (CEACAM1) induces angiogenesis of bladder cancer cells by increasing VEGF-C expression (25). Furthermore, the binding of VEGF-C to its co-receptor Neuropilin 2 (Nrp-2) on the lymphatic endothelium modulates developmental lymphangiogenesis and tumor metastasis (30).

However, not all responses mediated by VEGF-C are considered damaging. When expressed in appropriate levels in a chronic environment, there are considerable benefits. Through its role as a chemoattractant, VEGF-C has been shown to guide the beginnings of lymphatic vessel outgrowth by directing lymphatic endothelial cell migration into surrounding tissues, while in adult tissues, the VEGF-C signaling has been demonstrated to maintain lymphatic vessel integrity, promoting repair and regeneration during inflammation or tissue injury (31, 32). A study by D'Alessio et al. found that although VEGF-C alone strongly increased both M1 and M2 macrophage gene expression in mice with inflammatory bowel disease (IBD), anti-VEGFR3 antibodies aggravated inflammation and submucosal edema, increasing leukocyte infiltration (33). They postulated that VEGF-C exerts immunomodulatory effects through activation of the transcription factor STAT6 and resolving macrophages. Thus, a balance between pro-inflammatory M1 macrophages and anti-inflammatory resolving macrophages needs to be maintained when regulating VEGF-C expression. Similarly, severe lymphatic developmental defects have been observed in VEGF-C/VEGFR-3 knockout mice where the complete absence of lymphatic vessels sprouting and growth led to severe anemia, cardiac effusions and fluid accumulation in tissues (34).

### 3.3 The dichotomous role of lymphatic vasculature

At first glance, the lymphatic system plays seemingly opposing roles in inflammation and atherogenesis. While VEGF-C and other lymphagiogenic agents show benefit in the reduction of plaque inflammatory states, lymphatic hyperplasia and enhanced lymphangiogenesis is observed in a variety of inflammation-driven pathologies including psoriasis, ulcerative colitis, inflammatory arthritis, and even transplant rejections (23). There is a distinction between the protective anti-atherogenic state of the lymphatic system that guides resolution of inflammation, reverse cholesterol transport and immune cell egress with tolerance vs. the pathogenic pro-atherogenic functional state that leads to sustained chronic inflammation, perpetuating plaque inflammation and functional lymphatic remodeling within and around plaques. This duality relies heavily on the integrity of lymphatics, location, and temporal contexts.

Lymphatic integrity refers to the vessels functioning properly dictating their ability to efficiently transport lymph without leakage (35). Structural stability and functional capacity are the keystones in strengthening lymphatic systems. Structural support of non-leaky, intact endothelial junctions, can lead to efficient removal of plaque-producing cholesterol, cytokines, and immune cells all working to maintain vascular health and promote resolution of inflammation (36). Functionally, effective drainage, contractility and directional lymphatic flow helps clear inflammatory states (22). This system when damaged can lead to poor lymphatic drainage, immune cell and lipid retention, and cytokine accumulation all which fall into a pro-atherogenic state. Overall, healthy lymphatics can work to calm and clear inflammation while dysfunctional lymphatics hinder clearance and allow for worsening inflammation to develop.

Spatial context highlights the areas where lymphatic vessels may help in resolving inflammatory states vs. areas that may hinder clearance. Studies of mouse models of atherosclerosis have shown in early disease adventitia, enhanced lymphangiogenic states help drive cholesterol and immune cells away from the vessel promoting lipid and immune clearance and preventing progression of inflammatory states (37). When compared to pathologic lymphangiogenesis in spaces like advanced psoriasis, ulcerative colitis, and inflammatory arthritis, lymphangiogenesis is initially a compensatory response, but chronically it diverges from the intent and results in networks of ineffectual lymphatic drainage that sustains inflammation. This leads to persistent edema, accumulated cytokines, and inflammatory cells retained locally, along with antigen presentation cells becoming maladaptive all which fail to restore immune and fluid homeostasis (23).

As seen in the examples above, temporal context provides an additional layer in the factors of duality of when the lymphatic system can do more harm than good specifically when looking at acute vs. chronic phases. In acute phases, lymphatics can help promote immune cell exit, restore tissue homeostasis, and encourage immune tolerance all guiding towards inflammatory resolution (38). Comparatively, chronic, unresolved inflammation leads to continuous antigen presentation, persistent immune activation in draining lymph nodes and recycling of actuated T cells back to plaques leading down the path towards pathogenic pro-inflammatory states (39). In acute inflammation, lymphatic activation facilitates clearance of inflammatory mediators, whereas in chronic inflammation, persistent lymphatic remodeling promotes immune retention and ongoing inflammation. With the same system, there can be different outcomes depending upon disease stage and vessel integrity. The net effect of lymphatic vasculature is context-dependent, governed by vessel functionality, anatomical compartment, and the chronicity of inflammation.

### 3.4 Lymphatic regulation of atherosclerotic plaque progression and dysfunction as a driver

Functional lymphatic vessels play a critical role in regulating atherosclerotic plaque development by facilitating immune cell trafficking, lipid clearance, and resolution of vascular inflammation. Efficient lymphatic drainage from the arterial wall promotes the egress of macrophages and dendritic cells from atherosclerotic lesions to regional lymph nodes, limiting their prolonged retention within the intima. This process supports an anti-inflammatory immune milieu within plaques by favoring the presence of regulatory T cells (Tregs) and alternatively activated M2 macrophages (40). Tregs suppress excessive immune activation through the secretion of IL-10 and TGF-β, while M2 macrophages enhance efferocytosis, reduce necrotic core expansion, and promote fibrous cap stability through the secretion of profibrotic mediators such as IGF-1, fibronectin, and TGF-β (40–42). In parallel, lymphatic-mediated trafficking enables reverse cholesterol transport by mobilizing cholesterol-laden immune cells and lipids from plaques to draining lymph nodes and the systemic circulation for hepatic clearance. Collectively, these mechanisms limit plaque growth, dampen pro-inflammatory signaling, and promote plaque stabilization.

However, when lymphatic function is compromised, these protective mechanisms are lost, contributing directly to atherosclerotic progression. Structural and functional alterations in lymphatic vessels—including reduced contractility, impaired endothelial junction integrity, and disrupted chemokine signaling—diminish the capacity of lymphatics to drain inflamed arterial tissue. As a result, macrophages, dendritic cells, and foam cells become trapped within the vessel wall, sustaining chronic inflammation and promoting lipid accumulation (41). Impaired lymphatic drainage further disrupts reverse cholesterol transport, leading to cholesterol retention within plaques and expansion of unstable necrotic cores. Experimental studies demonstrate that aortic lymphatic transport deteriorates during atherosclerosis due to cholesterol-driven inflammation and arterial wall remodeling yet improves during lesion regression following lipid-lowering interventions such as ezetimibe therapy (43). These findings underscore a bidirectional relationship in which hyperlipidemia induces lymphatic dysfunction, while impaired lymphatic clearance perpetuates vascular inflammation and plaque vulnerability.

### 3.5 Lymphangiogenesis in the resolution of atherosclerosis

**Figure 4:**
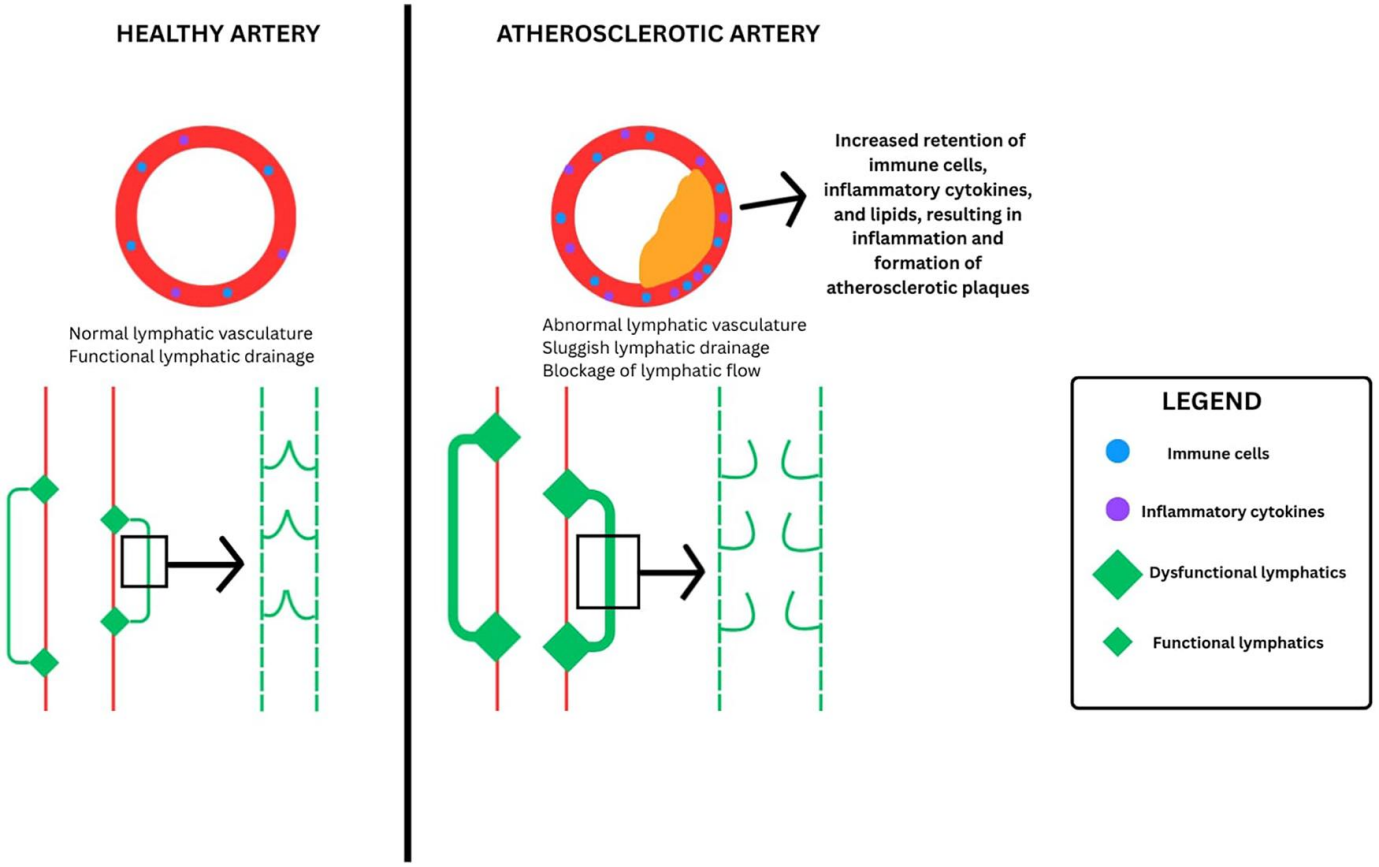
A figure summarizing the burden of atherosclerosis on the lymphatic system. Lymphatic dysfunction further contributes to plaque progression and impaired resolution of inflammation due to impaired clearance of lipids, immune cells, and inflammatory mediators from the arterial wall.

Resolution of atherosclerosis not only relies on lipid-lowering strategies, but on restoring lymphatic function, largely through lymphangiogenesis. Lymphangiogenesis plays a critical role in promoting cholesterol clearance through reverse cholesterol transport (i.e., removal of cholesterol from peripheral tissues), dampening inflammatory states, and re-establishing balance in vascular homeostasis (24, 44, 45). Impaired lymphatic drainage has been demonstrated to lead to defective cholesterol removal and unresolved inflammation in atherosclerosis models (46). Yeo et al. conducted a study where lymphangiogenesis was blocked via anti VEGFR3 antibodies in a mice aortic transplant model, resulting in a disruption of lymphatic drainage (38). In this model, reverse cholesterol transport was impaired, and cholesterol retention was seen within arterial plaques. Further correlation has been shown between lymphatic transport insufficiency and reduced lymphatic drainage of macromolecules, all which contribute to cholesterol and triglyceride accumulation in plaques and sustained vascular inflammation (Figure 4) (6, 47). Thus, improved lymphatic health and regrowth can not only facilitate movement of cholesterol in plaques to lymph nodes and ultimately to liver excretion, but it can also assist with resolving inflammation through the drainage of inflammatory cells, cytokines, apoptotic cells, and fluid, dampening as a result the effects of chronic vascular inflammation (46). Together, these actions lead to a decrease in chronic vascular inflammation that further hinders plaque progression and stability.

## 4 VEGF-C/VEGFR3 signaling as a lymphangiogenic therapy

Emerging therapies are increasingly recognizing the importance of the lymphatic system in vascular health, particularly in atherosclerosis management. VEGF-C has become the focus for several key therapies targeted against atherosclerosis. This has become evident as recent case studies such as the one by Byer et al. have indicated that VEGF inhibitors like bevacizumab may contribute to ischemic heart disease and accelerate atherosclerosis (48). This progression of atherosclerosis is partly attributed to reduced nitric oxide availability and decreased proliferation of endothelial cells (49). In their study, Silvestre-Roig et al. delivered VEGF-C attached to the F8 antibody (an antibody that targets the extra domain A of fibronectin, an isoform that accumulates in inflamed tissues) to atherosclerotic tissues in Apoe^−/−^ mice (50). They found that treatment did not significantly affect plasma cholesterol levels, circulating leukocyte counts, arterial lymph vessel area, and macrophage burden. However, they observed that F8-VEGF-C-treated mice had improved signs of plaque stability as exemplified by reduced necrotic core sizes, thicker fibrous caps, increased intimal smooth muscle cells (SMC), increased cholesterol efflux and reduced lipid accumulation in SMCs. This is important as atherosclerotic plaque destabilization is related to lipid toxicity.

Trials using VEGF-A, another key mediator of atherosclerosis that upregulates the migration of vascular SMCs into the atherosclerotic plaque, have demonstrated limited success. In 2009, the RadioNuclide imaging (NORTHERN) trial, a double-blind, placebo-controlled study, investigated the administration of vascular endothelial growth factor VEGF165, a VEGF-A isoform, in patients with refractory angina who were unsuitable for revascularization (51). The trial found no difference between the VEGF-treated and the placebo groups in improvement of anginal symptoms or change in myocardial perfusion from baseline to 3 or 6 months, the latter of which was assessed by single photon emission tomography (SPECT) imaging. This study could thus further suggest that VEGF-A is less potent in inducing lymphagiogenesis unlike VEGF-C, and more involved in the recruitment of monocytes and macrophages during inflammatory events (52).

### 4.1 Recombinant VEGF-C156S as a lymphangiogenic agent and a potential treatment for atherosclerosis

Recombinant human protein VEGF-C (VEGF-Cys156Ser or VEGF-C156S), a mutant variant of VEGF-C lacking VEGFR2 binding, has been seen to effectively promote lymphangiogenesis in preclinical animal models while avoiding unwanted angiogenesis via VEGFR2 activation. A 2024 systematic review by Fowler et al., examined multiple preclinical studies on VEGF-C156S, supporting evidence that this mutant protein selectively activates VEGFR3 (53). In a mouse model-based study by Karkkainen et al., recombinant VEGF-C156S administered via intraperitoneal injections was shown to effectively induce lymphangiogenesis without triggering blood vessel growth (54). This is further supported by the fact that VEGF-C156S has been shown to restore lymphatic vascular function leading to reduced inflammation and fibrosis (55). Similarly, a 2002 study by Szuba et al. found that administering a single subcutaneous dose of VEGF-C156S in a rabbit ear model significantly improved lymphatic drainage within a week (56). Although VEGF-C156S was designed specifically to selectively stimulate VEGFR3, which is the receptor considered specific to lymphangiogenesis, it is unknown whether the selectivity of only activating VEGFR3 is as beneficial as it is theorized (57). Visuri et al. showed that VEGF-C in its full form induces the preferred alternative for growth factor therapy of lymphedema when compared to VEGF-C156S, due to the robust lymphangiogenic response shown with VEGF-C (58). To this date, no direct trials and studies using VEGF-C156S have been conducted in terms of studying its effectiveness on targeting or stabilizing atherosclerotic plaques, future studies on VEGF-C156S could research on how improved pulmonary lymphatic drainage due to VEGF-C156S administration promotes lymphatic clearance of cholesterol and immune cells without exacerbating plaque growth and instability.

### 4.2 Nanoparticle formulations of oral VEGF-C and gene-based therapies

Building on these findings, therapies have further focused on the development of nanoformulations for VEGF-C delivery, especially the VEGF-Cys156Ser protein in the form of E-VEGF-C. A 2023 study by Juneja et al. in cirrhotic rats further demonstrated lymphatic function proliferation, improved drainage, and hemodynamic benefits from E-VEGF-C therapy (59). When it comes to atherosclerosis and atherogenesis, a recent study by Zhang et al. found that intramyocardial lipid nanoparticle (LNP) delivery of VEGF-C mRNA promoted VEGF-C secretion and lymphangiogenesis, reduced the infiltration of inflammatory cells, and inhibited pro-inflammatory and fibrosis in animal models following induction of a myocardial infarction (MI) (60). The study's findings highlighted its promising potential for clinical translation and the potential of lymphagiogenesis in dampening inflammatory responses. Thus, future research recommendations include optimizing delivery and targeting of platforms (e.g., tissue specific nanoparticles, fusion proteins like F8-VEGF-C) to further enhance lymphatic specificity and minimize systemic exposure (57, 58).

Similarly to recombinant protein strategies, gene therapies have demonstrated therapeutic potential of lymphangiogeneic enhancement. Gene therapy approaches (e.g., viral vectors, plasmids) carry risks of immunogenic responses, insertional mutagenesis, and variable expression across patients (61). Phase II clinical trials on gene therapies using an adenoviral vector to deliver VEGF-C gene directly into tissues have been initiated in human models of lymphedema (62). Lymfactin, a gene therapy that has entered Phase II trials, utilizes an adenovirus type 5–based vector to deliver full-length VEGF-C for breast cancer-related lymphedema (63). This therapy was engineered to promote endogenous VEGF-C expression, and lymphatic vessel formation and regeneration, particularly indicated for post-breast cancer surgery. Preliminary results have shown that lymfactin-treated patients exhibited greater reduction of subclinical interstitial fluid. Robust human data, however, on safety and efficacy in cardiovascular contexts nonetheless are lacking. Taking into account the lack of both preclinical and early phase clinical trials studying the effects of VEGF-C delivery for the treatment of atherosclerosis, the next progression of research would be to conduct long-term safety studies focused on vascular permeability, unintended angiogenesis, lymphatic overgrowth, and systemic immune effects.

## 5 Apolipoprotein A-I therapies

Apolipoprotein A-I (ApoA-I), the major protein component of high-density lipoprotein (HDL), is another emerging focus for novel therapies targeted against atherosclerosis. ApoA-I is commonly known for promoting reverse cholesterol transport (RCT) and regulating cholesterol trafficking by removing excess cholesterol from tissues, loading it into HDL particles, and delivering it back to the liver for excretion (64). ApoA-I boosts RCT via lymphatics by promoting ATP-Binding Cassette Transporter A1 (ABCA1). ABCA1 mediates cholesterol removal from macrophages and lymphatic endothelial cells, which not only reduces foam cell formation, but also suppresses inflammasome activation—both critical drivers of plaque formation. Because of this, ApoA-I's multiple mechanisms of action on lipid metabolism and inflammatory signaling creates synergistic anti-atherosclerotic effects (65, 66).

Recent research highlights a direct role for ApoA-I in the preservation of the integrity of lymphatic vessels. Beyond its commonly known function in RCT, this major protein component has been shown to support the stabilization of lymphatic endothelial junctions in collecting lymphatics. Stabilization of junctions further enhances lymphatic drainage of cholesterol and immune cells, further suggesting a broader anti-inflammatory effect in the reduction of arterial inflammation than previously understood (8). Studies have shown that treatment with lipid-free ApoA-I has been associated with improved lymphatic function, including reduced permeability and enhanced vessel contractility for improved clearance (8, 67). These findings highlight the therapeutic potential in modulating vascular inflammation by way of effects on lymphatic vessels.

Apart from improving the overall endothelial barrier function by steadying zipper-like junctions, ApoA-I further supports optimized lymph flow enhancing plaque regression by helping platelets interact with lymphatic endothelial cells (LECs) (8). The interaction between the glycoprotein podoplanin expressed on LECs and the C-Type lectin-like receptor 2 (CLEC-2) receptor expressed on platelets helps maintain the integrity of the lymphovenous junction, preventing backflow of blood into the lymphatic system. These actions encourage anti-inflammatory lymphatic function by preventing leaks and allowing for lymph outflow to effectively flow within the lymphatic vessel space (67). Collectively, these actions support ApoA-I as a promising therapy to restore lymphatic function in atherosclerosis. ApoA-I is integral in breaking the cycle of lipid accumulation and inflammation. This suggests it as a potential therapy targeting treatment atherosclerosis by improving lymphatic function.

### 5.1 ApoA-I infusions

ApoA-I infusions, including CSL112 (a formulation of purified ApoA-I), have gained attention as an emerging therapeutic strategy targeting atherosclerosis. Infusions have been designed to promote RCT by increasing functional ApoA-I and HDL particles aimed at enhancing RCT and reducing atherosclerotic burden (68, 69). CSL112 has been specifically created as a plasma-derived ApoA-I formation that promotes cholesterol efflux from macrophage foam cells, leading to reduced plaque lipid content and prevention of atherosclerotic lesion progression. A recent study from Mathias et al. showed that CSLF112 infusions accelerated RCT by elevating Lecithin–Cholesterol Acyltransferase (LCAT) enzyme levels in HDL particles and boosting cholesterol esterification and efflux (68). Another study on Ldlr−/− mice demonstrated that subcutaneous lipid-free ApoA-I infusion improved collecting lymphatic vessel integrity by reducing permeability, encouraging lymphatic transport and decreasing lipid and immune cell accumulation in atherosclerotic plaques (8).

Clinical trials on CSL112 have been conducted. In the AEGIS-II trial, 18,219 patients with acute MI, multivessel coronary artery disease, and additional cardiovascular (CV) risk factors were randomized to either four weekly infusions of 6 g CSL112 or placebo (70). The trial followed the patients over 90 days, 180 days, and 365 days. It found that CSL112 administration reduced the total burden of nonfatal ischemic events and CV death at 180 and 365 days compared with placebo, but it failed to reduce the primary end-point (a composite of myocardial infarction, stroke, or death from CV causes) at 90 days. However, a *post-hoc* analysis of the same trial revealed that, among patients treated with CSL12, those with LDL-C ≥ 100 mg/dL had statistically significant lower risk of recurrent cardiovascular events compared to patients with LDL-C levels lower than 100 mg/dL (70). These findings suggest that an individual's baseline cholesterol levels may influence therapeutic response, though prospective trials are needed to confirm this observation.

Other trials, albeit to a smaller extent, have investigated ApoA-1 infusions outside of CSL112. The MILANO-PILOT trial built upon prior trials that had demonstrated that ETC-216 administration, an ApoA-I Milano/phospholipid complex, in patients following acute coronary syndrome (ACS) showed promising atheroma regression (71). The trial studied MDCO-216, an updated and more immunologically inert formulation of ApoA-I Milano (72). It demonstrated that infusing MDCO-216 in statin-treated patients following ACS did not produce an incremental plaque regression. These findings parallel those of the CER-001 Atherosclerosis Regression Acute Coronary Syndrome Trial which investigated the use of CER-001, an HDL mimetic containing ApoA-1 and sphingomyelin, in statin-treated patients following ACS (73).

The outcome variability across ApoA-I infusions warrants further investigation. In summary, MDCO-216 and CER-001 showed no significant benefit in statin-treated patients following ACS (68, 69), while CSL112 demonstrated reduced total ischemic burden, particularly in patients with LDL-C ≥ 100 mg/dL (70). However, because all trials included patients with an LDL-C < 100 mg/dL, there are limiting conclusions about efficacy in patients with higher cholesterol levels. Moreover, when it comes to the translational relevance of ApoA-I infusions, it is important to note that they were used in patients with an increased atherosclerotic and inflammatory burden that was chronic and not acute. So while these therapies failed to produce meaningful results on an individual and a population level, on a cellular level, they contributed to a reduction in atheroma volume and increase in plasma levels of HDL-C. Therefore, these findings highlight that addressing atherosclerosis, and by extension any condition characterized by a high chronic inflammatory burden, needs to involve a multimodal regimen that targets multiple pathways and not just HDL functionality.

## 6 Macrophage-targeted therapies that could possibly enhance lymphatic-mediated inflammation resolution

Macrophages exist in several processes involved with the development of atherosclerosis. These include lipid uptake leading to foam cell creation and lesion formation, cholesterol storage through ACAT1-driven esterification, and impaired clearance of dead cells leading to a necrotic core formation and plaque vulnerability (74). Through the release of pro-inflammatory cytokines (e.g., TNF-a, IL-1β, IL-6, IL-8) they not only promote lesion progression, but also impair LEC junction integrity and lymphatic contractility while sustaining plaque inflammation (43). The interplay between macrophages and lymphatic vessels is central to coordinating immune responses and resolving inflammation. Targeting macrophages with inhibitors can allow for restoration of lymphatic vessel integrity and allow for better clearance of immune cells and pro-inflammatory molecules from arterial plaques. This highlights a novel therapeutic approach by addressing pathological states like atherosclerosis that are known to perpetuate chronic inflammation and hinder tissue repair through lymphatic dysfunction.

### 6.1 Therapeutic approaches to targeting macrophages

Inflammatory macrophages and NOD-like receptor family pyrin domain-containing 3 (NLRP3)/Interleukin-1β (IL-1β) signaling impair lymphatic vessel integrity- compromising lymphangiogenesis and drainage. The NLRP3 inflammasome is the sensor protein that triggers the release of cytokines IL-1β and IL-18, both of which drive chronic inflammation in atherosclerotic lesions and promote endothelial dysfunction (75, 76). Therapies aimed at suppressing inflammasome activity include canakinumab, a monoclonal antibody targeting IL-1β, which was evaluated in the CANTOS Phase III trial involving over 10,000 patients with prior myocardial infarction and elevated C-reactive protein (CRP). The trial showed that canakinumab significantly reduced recurrent cardiovascular events by approximately 15% (77). In the double-blind American RESCUE trial, 264 participants with moderate to severe chronic kidney disease (CKD) and high-sensitivity CRP were randomly allocated to subcutaneous administration of placebo or ziltivekimab, a fully human monoclonal antibody directed against the IL-6 ligand (78). The authors of the study reported that at 12 weeks after the randomization, CRP levels were reduced by 77% for the 7·5 mg group, 88% for the 15 mg group, and 92% for the 30 mg group. They stated that the results were stable at the 24-week follow-up and that subjects also exhibited dose-dependent reductions in fibrinogen, serum amyloid A, haptoglobin, secretory phospholipase A2, and lipoprotein(a), all of which play a crucial role in inflammation and lipid mediator syntheses. These findings were parallel to the findings in the RESCUE-2 trial that took place in Japan (79). The ongoing ZEUS trial will compare ziltivekimab to placebo among 6,200 patients with Stages 3–4 CKD and elevated CRP levels to investigate whether chronic IL-6 inhibition slows the progression of renal disease and reduces cardiovascular event (i.e., MI, stroke, CV death) rates (80).

A 2017 study on patients with psoriatic arthritis found that use of tumor necrosis factor inhibitors (TNFi) was associated with reduced progression of carotid plaques (assessed by carotid ultrasound after 2–3 years of use) in men and improvement in vascular inflammation (assessed by PET scan at 1 year) in both men and women (81). Similar findings have been reported in studies exploring CV events in patients with rheumatoid arthritis on TNFi (82). It is worth noting that the patients in the study suffered from autoimmune diseases, where the inflammatory burden is high. Nonetheless, chronic inflammation is a critical driver of CV disease, whose development may be accelerated in the presence of additional risk factors such as hypertension, smoking, and dyslipidemia (14). No clinical trials investigating IL-8 inhibitors have been conducted to this date; however, preclinical studies inhibiting IL-8 in animal models have shown promising results in downregulating pathways involved in atherosclerosis (83). Table 1 summarizes the findings of all of the aforementioned possible therapeutic investigations.

**Table 1:** Table summarizing investigational therapies targeting lymphatic function in atherosclerosis.

Therapeutic strategy	Mechanism of action/target pathway	Preclinical or clinical model	Key findings	Clinical status/limitations
VEGF-C/VEGFR3- Mediated Lymphangiogenesis	Enhances lymphatic vessel growth and drainage via VEGFR3 signaling; improves reverse cholesterol transport and reduces inflammation.	ApoE−/− mice with aortic plaques treated with F8-VEGF-C fusion protein.	↓ necrotic core size; ↑ fibrous cap thickness and smooth muscle cell content; ↑ cholesterol efflux without affecting plasma cholesterol.	Preclinical; requires validation in human models to assess vascular permeability and long-term safety.
Recombinant VEG-C156S	Selective VEGFR3 agonist promotes lymphangiogenesis without VEGFR2-mediated angiogenesis.	Mouse and rabbit models of lymphedema and inflammation.	Induced selective lymphatic growth; improved lymphatic drainage; ↓ tissue edema and inflammation.	No direct atherosclerosis trials; uncertain if VEGFR3-only activation yields optimal benefit.
Nanoparticle-Delivered VEGF-C (E-VEGF-C)	Encapsulation of VEGF-C156S in nanolipocarriers for targeted lymphatic delivery and sustain release.	Cirrhotic rat models; myocardial infarction (MI) mice.	↑ lymphatic proliferation; ↓ inflammation and fibrosis; improved drainage and cardiac function.	Promising for translation; delivery optimization and plaque-specific targeting needed.
Gene Therapy—Lympactin (Ad-VEGF-C)	Adenoviral vector-based VEGF-C gene delivery to promote endogenous lymphangiogenesis.	Phase II trials in breast cancer-related lymphedema.	↑ lymphatic vessel formation; ↓ interstitial fluid accumulation.	No cardiovascular or atherosclerosis trials; long-term safety and immune response data pending.
Apolipoprotein A-I (ApoA-I)	Promotes reverse cholesterol transport via ABCA1; stabilizes lymphatic endothelial junctions; reduces inflammation.	Ldlr−/− mice and human trials.	↓ foam cell formation; ↑ lymphatic contractility; ↓ vascular inflammation and permeability.	Effective in restoring lymphatic integrity; long-term impact on plaque regression in humans under investigation.
ApoA-I Infusions (CSL112)	Increases circulating functional HDL and ApoA-I to enhance cholesterol efflux.	AEGIS-II (Phase III; *n* = 18,219) post-MI patients.	↓ ischemic events and CV death at 180–365 days; benefit stronger in patients with LDL ≥ 100 mg/dL.	Safe and well-tolerated; uncertain optimal target population; no direct lymphatic metrics measured.
ApoA-I Mimetics (MDCO-216, CER-001)	HDL-mimetic peptides to enhance RCT and plaque regression.	MILANO-PILOT, CER-001 ACS trials.	No incremental plaque regression in low-LDL cohorts.	Limited efficacy: future studies needed in high-risk, high LDL groups.
Macrophage-Targeted Cytokine Inhibitors	Suppress inflammasome (NLRP3) and pro-inflammatory cytokines (IL-1β, IL-6, TNF-α) that impair lymphatic function.	CANTOS, RESCUE, RESCUE-2, ZEUS trials.	↓ CRP (up to 92%); ↓ recurrent CV events ∼15%; ↓ fibrinogen and lipoprotein(a).	Proven anti-inflammatory efficacy; direct lymphatic outcomes not yet studied.
TNF-α Inhibitors	Reduce systemic inflammation, improve endothelial and lymphatic function.	Psoriatic arthritis and RA cohorts (observational).	↓ carotid plaque progression; ↓ vascular inflammation (PET imaging).	Observational; mechanistic links to lymphatic repair require further validation.

### 6.2 Limitations and challenges of lymphagiogenic therapies in atherosclerosis

When it comes to therapies aiming to augment lymphagiogenesis, the most important thing to note is that there should be at least a degree of a normal functional lymphangiogenesis that can be augmented (84). Similarly, given that lymphagiogenesis and therapies aimed at treating atherosclerosis are heavily involved in reverse cholesterol transport, it is implied that HDL (whose levels are significantly higher in the lymph compared to blood) is largely intact (85). Nonetheless, HDL levels are commonly lower in patients with a known history of cardiovascular disease (86). Moreover, a lot of the studies that have been investigating therapies have not provided a 3D visualization of lymphatics or investigated the pumping rate of lymphangions, rendering the assessment of total lymph flow thus limited (85).

**Figure 5:**
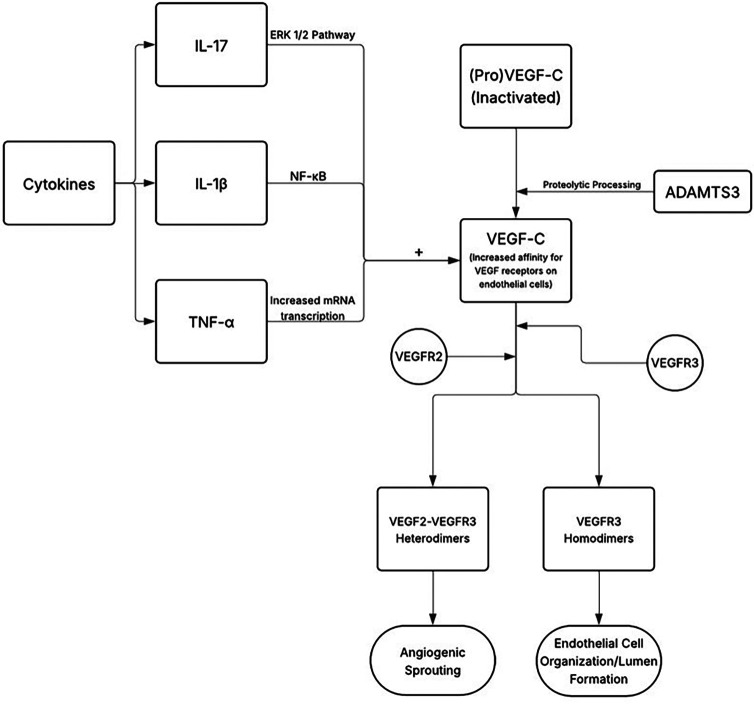
VEGF-C receptor signaling and downstream immunological effects.

Like previously mentioned, therapies relying on VEGF-C aim to primarily target VEGFR3 for lymphagiogenesis. However, VEGF-C has the ability to bind to other receptors like VEGFR2; such binding can stimulate angiogenesis and potentially limit the amount of VEGF-C for the growth of lymphatic endothelium (87). Thus, off-target effects may be observed as well (Figure 5). Adding to this argument, it is known that a lot of molecular factors which regulate lymphagiogenesis, are also involved in other functions. For example, angiopoietin 2 (Ang2) plays an important role in lymphatic development but it also contributes to the development of atherosclerosis by accelerating plaque rupture (85). Hence, adenoviral-mediated overexpression of Ang2 may theoretically lead to a larger size of atherosclerotic lesions at the expense of a better lymphatic vasculature. For this reason, researchers will need to carefully select which molecular factors to target to avoid these off-target effects. Finally, it is important to state that the time the lymphagiogenesis treatment is given in the disease course may be of some importance, as some preclinical studies have emphasized the importance of early treatment with VEGF therapies (6).

## 7 Clinical implications & future directions

Investigational therapies for atherosclerosis are increasingly extending beyond the arterial wall to consider the lymphatic system as a potential mechanistic mediator of vascular inflammation and durable plaque stabilization. A key question is whether reducing vascular inflammation improves lymphatic transport and drainage, enhances clearance of inflammatory mediators and cholesterol, and ultimately translates into clinically meaningful benefits. A few identifiable benefits being plaque regression, greater plaque stability, or fewer cardiovascular events. In this context, strategies that increase apolipoprotein-AI are being studied not only for their capacity to augment reverse cholesterol transport and attenuate arterial inflammation, but also for potentiating effects on lymphatic endothelial barrier function and cellular trafficking. In parallel, VEGF-C based approaches are under evaluation to promote lymphangiogenesis and improve lymphatic function. Anti-cytokine biologics, including IL-1β blockade with canakinumab and IL-6 pathway inhibition with tocilizumab, remain of interest as inflammation targeted interventions with previously established human safety experience. These medications may secondarily enhance lymphatic performance by reducing inflammatory signaling that impairs lymphatic contractility and endothelial function (88).

Although biomarker guided approaches may support patient selection, markers such as IL-6, TNF-α, and CRP are limited by substantial non-specificity. An indirect method to map lymphatic function involves identifying markers for LECs. These include the marker genes prospero homeobox 1 (Prox1) and Fms related receptor tyrosine kinase 4 (Flt4), the atypical chemokine receptor ACKR4 for ceiling LECs (cLECs) and the mucosal vascular addressin cell adhesion molecule 1 (MADCAM1) and lymphatic vessel endothelial hyaluronan receptor 1 (LYVE1) for floor-lining LECs (fLECs) (89). LECs also play a crucial role in lymphatic permeability and antigen presentation to T-cells by expressing high levels of the inhibitory receptor PD-L1 (90). They further attract dendritic cells through the production of various chemokines, mainly CCL21, which along with CCL19 assists in the recruitment of naive T cells into lymph nodes. While this is just a snapshot of some lymphatic-specific biomarkers, they highlight the need to shift away from more commonly used markers that are shared among multiple inflammatory pathways, and move towards the use of more specific markers that could be used to characterize the lymphatic function of patients and their response to the aforementioned therapies.

Furthermore, a major barrier to translating lymphatic targeted approaches into clinical atherosclerosis trials is the lack of robust, validated measures of lymphatic structure and function that can be applied at scale. Circulating extracellular vesicles (EVs) are emerging as potential translational biomarkers that may reflect lymphatic vessel health, endothelial activation, and immune trafficking. They are released by cells upon their activation or death, with accumulation of EVs in atherosclerotic lesions being suggestive of poor lymphatic clearance (91). Advances in lymphatic imaging, including near infrared fluorescence lymphography, MR lymphangiography, CT lymphangiography, and lymphoscintigraphy, now permit increasingly detailed assessment of lymphatic anatomy and transport dynamics in both preclinical and clinical settings (92–96). Integrating biomarker and imaging endpoints will be critical for patient stratification, mechanistic validation, and monitoring of therapeutic response, particularly as lymphatic focused interventions move toward combination strategies with lipid lowering and anti-inflammatory therapies.

Given this interplay between lipid metabolism, inflammation, cardiovascular disease, and lymphatic dysfunction, combination strategies that address both lipid burden and inflammatory signaling may offer meaningful advantages over single pathway approaches. Integrating lipid lowering therapies such as statins, ezetimibe, or PCSK9 inhibitors with cytokine targeted agents could synergistically reduce atherosclerotic burden while supporting lymphatic vessel health by limiting lipid accumulation, reducing inflammatory injury, and improving reverse cholesterol transport. Preclinical studies further suggest that intact aortic lymphatic drainage is required for ezetimibe driven atherosclerosis regression and that lymphatic directed interventions can amplify the benefits of lipid lowering and anti-inflammatory therapies (37, 43, 97–101). This data supports a dual targeted framework for complex disease states in which lymphatic inflammation and impaired clearance contribute to progression. At the same time, translation will require careful attention to safety, particularly the risk of additive immunosuppression and unintended effects on lymphangiogenesis, as well as the need for biomarkers that can more specifically track lymphatic function and treatment response.

However, as of mid-2025, there are no published or registered human clinical trials in atherosclerosis that specifically incorporate lymphatic endpoints or combine lymphatic-targeted therapies with standard lipid-lowering or anti-inflammatory agents. The field is poised for translational advancement, with several promising therapeutic strategies and drug delivery platforms nearing readiness for first-in-human studies. The integration of lymphatic endpoints and the combination of lymphatic-targeted therapies with standard agents represent important future directions.

## 8 Conclusion

The current state of translational and clinical evidence strongly supports the therapeutic potential of targeting vascular lymphatic dysfunction in atherosclerosis. VEGF-C/VEGFR3 agonists, anti-cytokine biologics, and advanced macrophage-targeted nanoplatforms have demonstrated robust efficacy in preclinical models, with several agents possessing established human safety profiles in other indications. Quantitative outcomes in animal studies include reductions in plaque area of and macrophage content along with reduction in inflammatory biomarkers. Human data are nonetheless limited to observational studies, biomarker analyses, and clinical trials of anti-cytokine therapies, with no published trials directly targeting lymphatic dysfunction in atherosclerosis as of mid-2025.

Immediate actionable steps for clinical translation include the initiation of early-phase human trials with VEGF-C-based agents, integration of lymphatic biomarkers and imaging modalities for patient selection, and careful monitoring of safety and efficacy endpoints. Patient subgroups most likely to benefit include those with early or subclinical atherosclerosis, genetic or metabolic predisposition to lymphatic dysfunction, chronic inflammatory comorbidities, and imaging or biomarker evidence of impaired lymphatic drainage. Combination strategies with standard lipid-lowering and anti-inflammatory agents are expected to be synergistic but require rigorous evaluation for potential adverse interactions.

In summary, the field is on the cusp of translating decades of mechanistic and preclinical research into clinical application. The integration of lymphatic-targeted therapies with established cardiovascular treatments holds promise for addressing residual risk and improving outcomes across the spectrum of atherosclerotic disease. Ongoing research should prioritize the design of robust clinical trials, validation of diagnostic and monitoring tools, and the development of precision medicine approaches tailored to individual patient characteristics and disease stage.
